# Remobilisation of phosphorus fractions in rice flag leaves during grain filling: Implications for photosynthesis and grain yields

**DOI:** 10.1371/journal.pone.0187521

**Published:** 2017-11-02

**Authors:** Kwanho Jeong, Cecile C. Julia, Daniel L. E. Waters, Omar Pantoja, Matthias Wissuwa, Sigrid Heuer, Lei Liu, Terry J. Rose

**Affiliations:** 1 Southern Cross Plant Science, Southern Cross University, Lismore NSW, Australia; 2 Southern Cross GeoScience, Southern Cross University, Lismore NSW, Australia; 3 Instituto de Biotecnología, Universidad Nacional Autónoma de México, Apdo., Cuernavaca, Morelos, Mexico; 4 Crop, Livestock and Environment Division, Japan International Research Center for Agriculture Science, 1–1 Ohwashi, Tsukuba, Ibaraki, Japan; 5 Department of Plant Biology and Crop Sciences, Rothamsted Research, West Common, Harpenden, Herts, United Kingdom; Louisiana State University College of Agriculture, UNITED STATES

## Abstract

Phosphorus (P) is translocated from vegetative tissues to developing seeds during senescence in annual crop plants, but the impact of this P mobilisation on photosynthesis and plant performance is poorly understood. This study investigated rice (*Oryza sativa* L.) flag leaf photosynthesis and P remobilisation in a hydroponic study where P was either supplied until maturity or withdrawn permanently from the nutrient solution at anthesis, 8 days after anthesis (DAA) or 16 DAA. Prior to anthesis, plants received either the minimum level of P in nutrient solution required to achieve maximum grain yield (‘adequate P treatment’), or received luxury levels of P in the nutrient solution (‘luxury P treatment’). Flag leaf photosynthesis was impaired at 16 DAA when P was withdrawn at anthesis or 8 DAA under adequate P supply but only when P was withdrawn at anthesis under luxury P supply. Ultimately, reduced photosynthesis did not translate into grain yield reductions. There was some evidence plants remobilised less essential P pools (e.g. Pi) or replaceable P pools (e.g. phospholipid-P) prior to remobilisation of P in pools critical to leaf function such as nucleic acid-P and cytosolic Pi. Competition for P between vegetative tissues and developing grains can impair photosynthesis when P supply is withdrawn during early grain filling. A reduction in the P sink strength of grains by genetic manipulation may enable leaves to sustain high rates of photosynthesis until the later stages of grain filling.

## Introduction

Phosphorus (P) is a key component of primary metabolites such as ATP, nucleic acids and phospholipids that are critical to central metabolism [1]. Phosphorus cannot be substituted by any other element [2], making it essential for plant growth and development. Phosphorus plays a vital role in energy metabolism and metabolic regulation, and is the essential substrate for phosphorylation during the production of ATP from ADP, the production and export of triose-P and the regeneration of ribulose-1,5-biophosphate (RuBP) during photosynthesis[3–5].

During senescence in annual crop plants, P is mobilised from leaves and other vegetative tissues and translocated to developing seeds, which are a strong P sink during the reproductive growth phase [6–8]. Senescence results in the breakdown of chlorophyll and reductions in photosynthetic activity which has been linked to the remobilisation of nutrients–particularly nitrogen (N)–from the leaves [9]. The link between P nutrition and photosynthetic capacity has also been well documented [10, 11], and it is therefore possible that remobilisation of P from leaves during senescence may be detrimental to leaf function, and any reduction in photosynthesis may have consequences for assimilate availability during grain filling. Indeed, Grabau et al. [12] demonstrated in soybean (*Glycine max*) that increased P nutrition during seed filling led to delayed leaf senescence and higher grain yield, and conversely, the withdrawal of P from the nutrient solution during seed filling in canola (*Brassica napus*) led to a reduction in plant biomass and seed yield [13]. The premise of these studies was that developing seeds are strong sinks for P, and the remobilisation of P from vegetative tissues results in insufficient P for other processes necessary for continued growth such as photosynthesis.

In rice (*Oryza sativa* L.), P is predominantly mobilised from leaves during the later stages of grain filling [14, 15] and a number of genes potentially involved in the regulation and translocation of P from senescing rice flag leaves have recently been identified [16]. Mobilisation of P from rice leaves to grains correlates with leaf senescence [17], but the specific impact of P remobilisation during grain filling on leaf photosynthesis is unclear. While rice is typically cultivated as an annual crop plant, most cultivars have retained a degree of perenniality so that, unlike other annual small grain crops like wheat (*Triticum aesitvum*), P uptake from the soil continues in rice until maturity [18]. What is not known is whether this late P uptake is crucial to maintaining high yield as it may delay senescence, or whether it merely provides further P to be stored in the seed, presumably for the benefit of developing seedlings.

Phosphorus pools in plant tissues can be broadly divided into four major groups, which are nucleic acid-P (P in RNA and DNA), structural-P (lipid-P such as membrane phospholipids), metabolic-P (inorganic P [Pi] and sugar phosphates such as ATP) and residual-P (other P compounds that do not fall into any of the previous categories) [19]. The metabolic-P group may play an important role in P remobilisation, because it contains the most mobile P fractions. The metabolically active Pi pool is located in the cytosol of plant cells within relatively narrow limits [20, 21], and any excess Pi is stored in the vacuole, which functions as a buffer to maintain Pi concentrations in the cytosol [22], while the sugar phosphates pool is used as intermediates in carbon metabolism such as in the calvin cycle and glycolysis.

The impact of P remobilisation from leaves to seed on leaf photosynthesis is not known. While it seems logical that the P depletion of leaves occurring post-flowering would impair leaf photosynthesis, it is also likely that P pools that are less essential for critical leaf functions may be remobilised first to retain P involved in key processes in leaves for as long as possible. In particular, vacuolar Pi may be mobilised first, given it is mobile and not critical for cellular function, and phospholipid-P may be remobilised before either nucleic acid-P or cytosolic Pi, as suggested by the upregulation of genes involved in replacement of phospholipids with other lipids in rice flag leaves during grain filling [16]. Hence, phospholipids could effectively be remobilised with little impact on photosynthesis, as is observed in highly P-efficient proteaceous plants [23].

Given the essential role of flag leaves in plant nutrient remobilisation and assimilation of carbon through photosynthesis during grain filling in cereals, in our study, we have focused on flag leaves.

The objective of this study was to test the following hypotheses:

1. Reduced P uptake after anthesis will lead to reductions in flag leaf photosynthesis, possibly as a result of earlier senescence;
2. Any reduction in flag leaf photosynthesis would lead to reduced plant biomass production, with consequences for grain yields;
3. Plants are selective in terms of the cellular P fractions that are remobilised which, to maintain photosynthesis, results in sequential mobilisation of P from the least to most essential P pools.

## Materials and methods

### Preliminary experiment to determine appropriate P rates for adequate and luxury P supply

A preliminary experiment was conducted to determine ‘adequate’ P supply, which was defined as the minimum amount of P needed to achieve maximum grain yields.

Rice (cv. IR64) seeds were sterilised using HClO_3_ for 2 min and germinated in Petri dishes in the dark at 30°C for 2 d. Germinated seeds were transferred to mesh floating above a solution containing 1 mM calcium (CaCl_2_) and 36 μM iron (Fe EDTA). After 10 d the solution was changed to half strength Yoshida solution [24] without P, in which the plants were grown for a further 2 weeks, with nutrient solution replaced after 1 week. Two equal sized seedlings were then transplanted into 5 L containers wrapped with aluminium foil containing full strength Yoshida solution without P. Nutrient solution was changed every week and the pH of the solution adjusted to 5.2–5.5. Plants were grown under temperature-controlled conditions in a glasshouse at Southern Cross University (Lismore, NSW, Australia) with a mean day/night air temperature of 29°C/21°C and relative humidity (RH) of 75%.

In order to determine the P rate at which maximum biomass and grain yield were achieved, ten P treatments were supplied to ten groups of three replicate containers at the rates of 0.3, 0.45, 0.6, 0.675, 0.75, 0.825, 0.9, 0.975, 1.2 and 1.5 mg of P (as KH_2_PO_4_) per day per container by applying to the nutrient solution the appropriate volume of P stock solution (150 mg P L^-1^) every 3.5 days until plants were matured.

At maturity (124 days after germination), plants were removed from containers, separated into grain, straw and root and dried for 7 d at 40°C in a drying room. Total grain weight (TGW) and dry weight (DW) of total aboveground plant were plotted against P treatment and fitted by a segmented linear regression (“broken stick regression”) using R [25]. This resulted in two segments of linear regression (Segment 1 and Segment 2) described by the two following equations:

$$\begin{array}{ll} y=a_{1}x+b_{1} & \;for\;x\leq BP-\mathrm{Segment}1\\ y=a_{2}x+b_{2} & \;for\;x\geq BP-\mathrm{Segment}2 \end{array}$$

where *x* is the P treatment, *y* is the response variable (TGW or DW), *a*_*i*_ and *b*_*i*_ are the slope and the intercept at y-axis of linear segment *i* (1 ≤ i ≤ 2), *BP*_*i*_ is the breakpoint of linear segment *i* (value of x which verifies both equations).

### Phosphorus withdrawal experiment

To determine the impact of competition for P between vegetative tissues and developing seeds during grain filling, P was permanently withdrawn from the nutrient solution at three time points during grain filling while supplied until maturity in the control with either adequate or luxury P applied to the nutrient solution. Previous experiments have indicated that the pattern of P mobilisation in flag leaves during grain filling is similar to that of the lower leaves; hence, measurements in the present study were taken on the flag leaf [14].

Rice seeds (cv. IR64) were germinated and grown on a mesh for 2 weeks as described above. Seedlings were then transferred to 5 L containers as above containing full strength Yoshida solution without P. Two P treatments were established to create plants grown with adequate P supply and luxury P supply. The P rates required to achieve this were 0.75 mg P day^-1^ (adequate P) and 1.5 mg P day^-1^ (luxury P) based on results of the preliminary experiment.

Three P-restriction treatments were imposed: P was permanently withdrawn from the nutrient solution at anthesis (0 DAA) (T1), at 8 DAA (T2) or 16 DAA (T3), while in the control treatment, P was continually supplied in the nutrient solution until maturity at 30 DAA as a control (C). Each P supply level (adequate or luxury) x P treatment (T1-T3, C) combination was replicated three times. Anthesis was defined as when 50% of panicles had at least 50% of florets with anthers visible. In all treatments (T1-T3, C) at both P levels (adequate and luxury), a set of six panicles that reached booting at the same time were tagged for future measurements of photosynthetic activity and leaf P fractions (see below).

### Photosynthetic rate measurements

Net photosynthetic rate was measured on six flag leaves per container using a LI-6400XT Portable Photosynthesis System (LI-COR Biosciences, Lincoln, USA) under 2000 μmol m^-2^ s^-1^ photon flux density with a 6400–02 LED light source and 400 μmol / mol CO_2_. The temperature and flow were set at 27°C and 500 μmol s^-1^, respectively. The data were recorded on flag leaves at five times: at booting, anthesis (0 DAA), 8 DAA, 16 DAA and maturity (30 DAA). Data were logged when CO_2_, H_2_O and flow values were stable.

After measuring net photosynthetic rate, the six flag leaves from tagged panicles per container were harvested, pooled and immediately frozen in liquid nitrogen and kept in -80°C for later P fraction analysis.

### Rice flag leaf total P and P fraction measurements

To determine P concentrations of the different P fractions, the frozen flag leaf samples were ground in liquid nitrogen using a mortar and pestle and freeze dried for 48 h. Three subsamples of 25 mg of the freeze-dried flag leaf samples were processed to measure and all measurement was proceeded with three biological replicates:

1. The total P concentration
  A freeze-dried flag leaf subsample was digested with 2.5 ml of nitric acid (HNO_3_) using a MARS Xpress microwave oven (CEM Corporation, North Carolina, USA). After digestion, each sample was diluted to a final volume of 10 ml with Milli-Q water and P concentration measured by inductively coupled plasma mass spectrometry (ICP-MS) (Perkin Elmer, Massachusetts, USA)
2. Pi concentration
  A freeze-dried flag leaf subsample was extracted with 1 ml of 0.4 M HCl for 3 h at room temperature. This extract was diluted with Milli-Q water to a total volume of 25 ml and Pi was measured using the molybdate and malachite green assay (Motomizu et al. 1983). Sample absorbance was measured at 650 nm using an MWG Sirius Plate reader (MWG Biotech, Ebersberg, Germany), and Pi concentration calculated by normalisation of dry weight (mg).
3. Organic P fractions concentrations
  The P containing compounds of the freeze-dried flag leaf subsample were fractionated into the following four fractions; lipid-P (i.e. phospholipids), metabolic-P (easily soluble P-containing metabolites such as ATP and sugar phosphates), nucleic acid-P (P in RNA and DNA) and residual-P (phosphoproteins and unidentified residue) following a modified fractionation assay from Hidaka and Kitayama [19]. Freeze dried flag leaf subsample was weighed into a 2 ml screw cap vial and extracted three times with 1 ml of a mix of chloroform: methanol: formic acid (CMF, 12:6:1 v/v/v, 3 ml total per sample). The CMF extracted leaf residue was then extracted three times with chloroform: methanol: water (CMW, 1:2:0.8, v/v/v, total of 3.78 ml of CMW per sample). This extract was transferred to a 22 ml glass tube and 1.9 ml of chloroform washed water (10% of miliQ water + 90% of chloroform) was added. This extract solution was mixed and partitioned into a lipid-rich organic (bottom) layer and a sugar- and nutrient-rich aqueous (upper) layer. The aqueous layer was transferred carefully into a 22 ml glass tube (Fraction 2a) and the bottom chloroform layer was dried under N (Fraction 1, lipid-P). After the CMF and CMW extraction, the leaf residue was washed with 1 ml of 85% methanol (Fraction 2b). After methanol extraction the residue was dried under N to remove the methanol, and then extracted twice with 1 ml of cold 5% trichloroacetic acid (TCA, 4°C). Each extraction was for 1 h at 4°C, the sample was mixed by inversion every 10 min and a total of 2 ml of 5% TCA was used for each sample (Fraction 2c). Fractions 2a, 2b and 2c were combined and taken into a new 22 ml glass tube to form the final Fraction 2 (metabolic-P). The leaf residue was again extracted with 1 ml of 2.5% TCA, three times at 95°C for 1 h, a total of 3 ml 2.5% TCA supernatant was taken into a new 22 ml glass tube (Fraction 3; nucleic-P). Fraction 2 and 3 were dried in a rotary vacuum concentrator (Martin Christ, Osterode am Harz, Germany) for 4 h at 50°C. The extracted residue was dried under N (Fraction 4, residue-P). To measure the P concentration of each fraction, each dried fraction was digested with 2.5 ml of HNO_3_ using the MARS Xpress microwave oven. After digestion, each sample was diluted to a final volume of 10 ml with Milli-Q water and P concentration measured by ICP-MS. More details of the P fraction assay procedure can be found in S1 Fig.

We separated Pi from other metabolic-P such as ATP and sugar-P. Consequently, in our study, metabolic-P refers to metabolic-P without Pi.

### Yield components, tissue biomass and P concentrations

At maturity (30 DAA), whole plants were harvested and the total number of productive main tillers, late tillers, TGW and total biomass recorded. Harvested plants were separated into root (R), stem (S), flag leaf (FL), leaf (L), dead leaf (DL), late tillers (LT) and panicle (PC). Separated samples were then dried for 7 d at 40°C in a drying room. A leaf was considered dead when more than 70% of its area was yellow and LT referred to those tillers that had not yet reached flowering. Following drying, panicles were separated into husk (H), grain (G) and rachis (RH). Dry weight of all tissue samples were measured. The total P concentration of each tissue sample (~ 0.2 g) was measured using the same method as for flag leaves except 5 ml of HNO_3_ was used and diluted to a final volume of 25 ml with Milli-Q water (see above). Phosphorus content was calculated by multiplying P concentration by the dry weight. Harvest index (HI) was calculated by TGW divided by the total aboveground plant DW and Phosphorus harvest index (PHI) as P content of whole grain (grain + husk) divided by the P content of total aboveground plant tissues.

### Statistical analyses

All statistical analyses were performed with Genstat version 16.1 [26]. All yield component, biomass and P data were analysed using a two-way ANOVA fitting time of P withdrawal (0 DAA, 8 DAA, 16 DAA and 30 DAA [control]) and P supply level (adequate or luxury) as fixed factors. For flag leaf P fraction and photosynthesis data, adequate P supply and luxury P supply data were analysed separately. For each P supply level, flag leaf P fraction and photosynthesis data in the control treatment (P supplied until maturity at 30 DAA) were analysed using a one-way ANOVA fitting time of measurement (booting, anthesis [0 DAA], 8 DAA, 16 DAA and 30 DAA) as the fixed factor. Within a given time of measurement, flag leaf P fraction and photosynthesis data were analysed using a one-way ANOVA fitting time of P withdrawal (0 DAA, 8 DAA, 16 DAA and 30 DAA [control]) as the fixed factor. Significance of differences between treatment means for each parameter were tested using Duncan’s multiple range test (*P* ≤ 0.05).

## Results

### Preliminary experiment to determine appropriate P rates for adequate and luxury P supply

**Fig 1:**
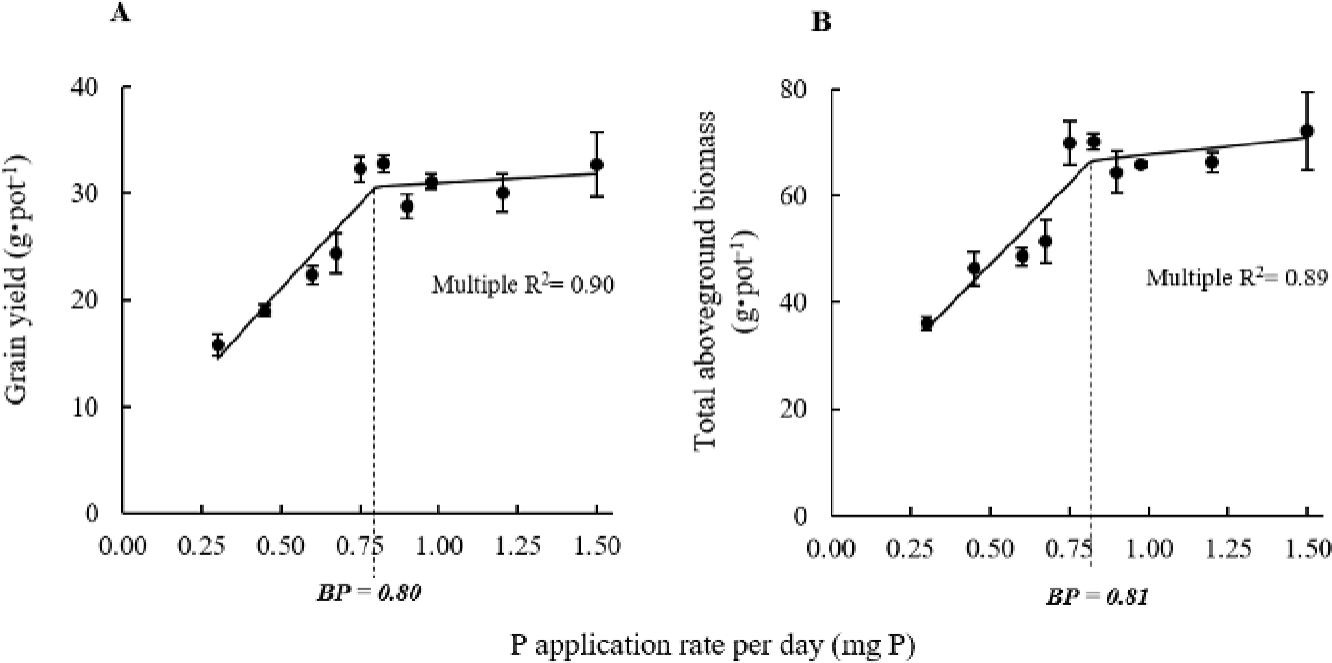
**Grain yield (A) and total aboveground biomass (B) of hydroponic rice plants in response to increasing phosphorus supply in the nutrient solution.** Black solid lines represent the segmented linear regressions and values in bold italic are the estimated breaking point (BP).

In both linear regressions on TGW and DW, linear “Segment 1” was characterised by an increasing response to P treatment whereas linear “Segment 2” showed little response to P treatment and indicated the yield plateau. At the junction between both segments, the breakpoint thus represented the P treatment at which maximum response was achieved. In both linear regressions, the estimated breakpoint was 0.8 mg P per day (overall R^2^ ≥ 0.89) (Fig 1A and 1B). For the subsequent experiments, P-application rates comprised between 0.75 and 0.85 mg P per day and it was thus considered adequate P treatment and any P-application rate well above 0.85 mg P per day was considered a “luxury” P treatment.

### Impact of restricted P supply during grain filling on yield, P remobilisation from the flag leaf and flag leaf photosynthesis

#### Effect on plant growth and yield components

The level of P supplied in the nutrient solution (adequate or luxury) had a significant effect on most phenotypic traits measured at maturity, with higher productive tiller numbers, total biomass and grain weight and grain P concentration under luxury P supply compared with that at adequate P supply (P ≤ 0.05; Table 1). Interestingly, grain yield was higher at the luxury P level when P was withdrawn from the nutrient solution at anthesis (T1) than that in all other treatment combinations (Table 1).

**Table 1:** The impact of P supply level (adequate or luxury) and time of P withdrawal during grain filling on key traits at maturity. Means within a column not followed by a common letter are significantly different at *P* ≤ 0.05. T1; P was withdrawn at anthesis, T2; P was withdrawn at 8 DAA, T3; P was withdrawn at 16 DAA, control; P was supplied until maturity.

P withdrawal treatment	P supply level	Number of productive main tillers	Number of late tillers	Total grain weight (g)	Total biomass (g)	Total aboveground plant P content (mg)	Grain P concentration (mg g^-1^)	HI	PHI
T1	Ade P	10.67 ^bc^	1.5 ^ab^	40.41 ^a^	119.95 ^ab^	50 ^a^	1.249 ^a^	0.417 ^b^	0.679 ^cd^
	Lux P	12.83 ^c^	2 ^ab^	49.99 ^b^	148.53 ^c^	87.67 ^c^	1.672 ^b^	0.401 ^b^	0.653 ^bcd^
T2	Ade P	10.33 ^ab^	1 ^ab^	37.38 ^a^	112.42 ^ab^	49.21 ^a^	1.243 ^a^	0.408 ^b^	0.632 ^abcd^
	Lux P	11.33 ^bc^	2.67 ^b^	41.41 ^a^	116.87 ^ab^	88.78 ^c^	2.052 ^c^	0.423 ^b^	0.674 ^cd^
T3	Ade P	8.33 ^a^	1.17 ^ab^	32.69 ^a^	96.77 ^a^	55.70 ^a^	1.694 ^b^	0.413 ^b^	0.678 ^d^
	Lux P	9.67 ^ab^	4.5 ^c^	33.43 ^a^	110.86 ^ab^	105.41 ^d^	2.802 ^d^	0.353 ^a^	0.618 ^abc^
Control	Ade P	10.5 ^ab^	0.83 ^a^	35.52 ^a^	113.63 ^ab^	66.65 ^b^	1.672 ^b^	0.381 ^ab^	0.611 ^ab^
	Lux P	11 ^bc^	4.5 ^c^	41.25 ^a^	130.82 ^bc^	128.78 ^e^	2.622 ^d^	0.379 ^ab^	0.588 ^a^
P withdrawal treatment		< 0.01	< 0.001	< 0.004	< 0.02	< 0.001	< 0.001	< 0.044	< 0.011
P supply level		< 0.024	ns	< 0.018	< 0.023	< 0.001	< 0.001	ns	ns
Interaction		ns	< 0.029	ns	ns	< 0.001	< 0.044	ns	ns

The timing of P withdrawal from the nutrient solution had a significant effect on tiller numbers, biomass and grain yields, total aboveground plant P content, grain P concentrations and HI and PHI (P ≤ 0.05; Table 1). The withdrawal of P from the nutrient solution at either anthesis (T1) or at 8 DAA (T2) led to a significant (P ≤ 0.05) reduction in late tillers (2 and 2.67, respectively) compared with that in the T3 and control (C) (4.5 late tillers) in the luxury P treatment, while only 1–2 late tillers were observed in the adequate P supply treatment regardless of the time of P withdrawal from the nutrient solution. Interestingly, while grain P concentrations tended to be lower when P was withdrawn from the nutrient solution at anthesis (T1) or 8 DAA (T2), the proportion of total P in the grains tended to be higher, with a trend of increasing PHI with earlier withdrawal of P from the nutrient solution (Table 1).

#### Effect on P partitioning among tissues at maturity

**Fig 2:**
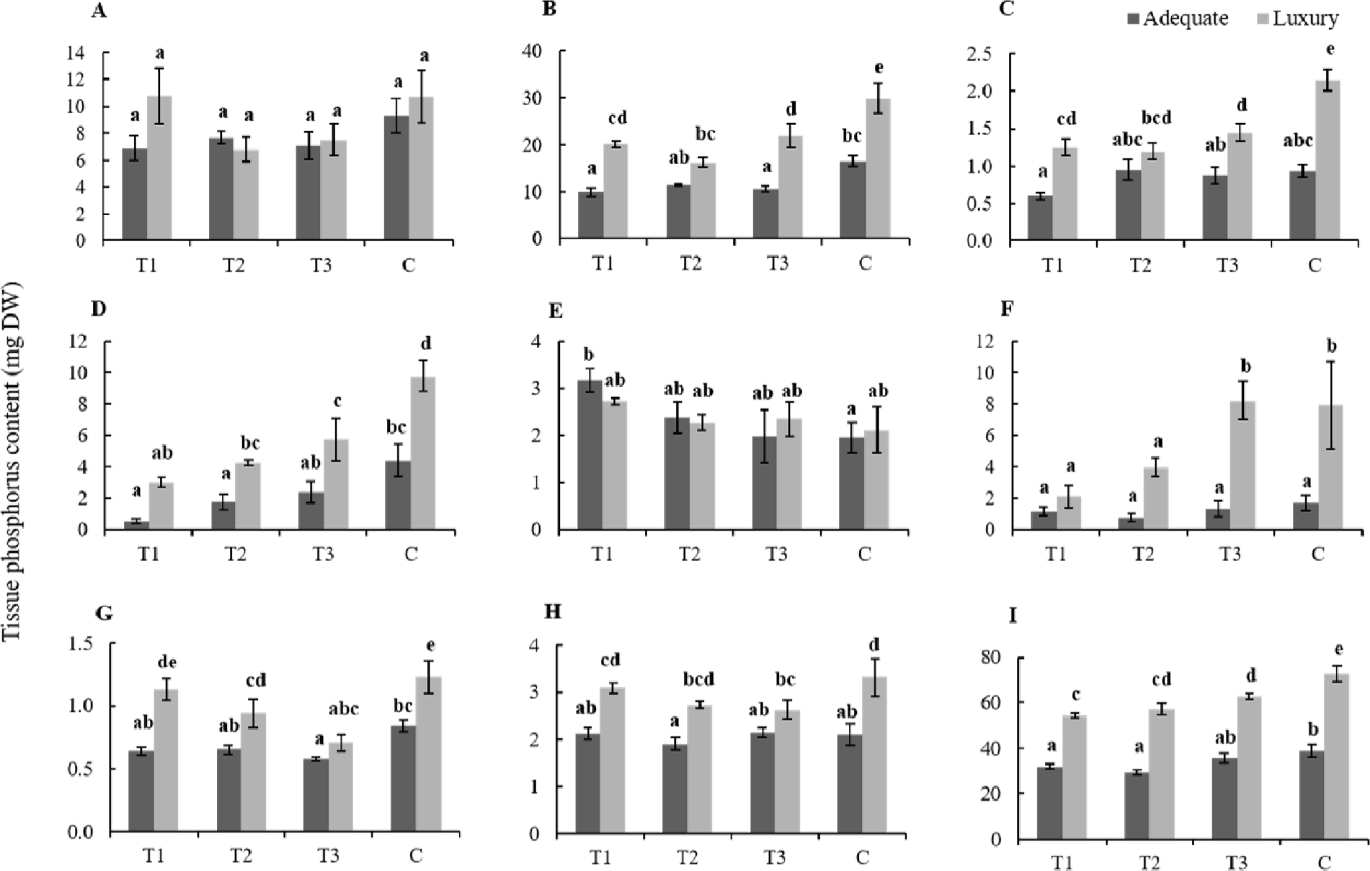
**Phosphorus contents rice root (A), stem (B), flag leaf (C), leaf (D), dead leaf (E), late tillers (F), rachis (G), husk (H) and grain (I) at maturity (30 DAA) in T1, T2, T3 and control plants (C) of both adequate and luxury P treatments.** Bars represent SEM (n = 3). Columns that do not share a common letter are significantly different at P ≤ 0.05 (n = 3).

As expected, P contents at maturity were higher in the luxury P treatment in all tissues except roots and fully senesced (dead) leaves (Fig 2; *c*.*f*. y-axis scales are different), which contained around 8–10 mg P container^-1^ and 2–3 mg P container^-1^, respectively, regardless of P supply level or the timing of P withdrawal from the nutrient solution (Fig 2A and 2E). Under luxury P supply, generally the longer P was supplied in the nutrient solution the higher the content of P in flag leaves, leaves, stems, late tillers and grains at maturity. In contrast, the contents of P in root, dead leaves, rachis and husk were typically unaffected by the timing of withdrawal of P from the nutrient solution under luxury P supply (Fig 2A, 2E, 2G and 2H). Under adequate P supply, a clear trend in increased P content with longer duration of P supply was only observed in the leaves (Fig 2D), with few significant differences due to P withdrawal observed in other tissue types. Ultimately, the grain was the main P sink during grain filling (Fig 2I).

#### Effect on remobilisation of P from different P fractions in flag leaves

Recovery of P from the fractionation procedure ranged from 95–105% (S2 Fig) and concentrations of P in the residual P fraction (Fraction 4) were less than 0.07 mg g^-1^ for all P withdrawal treatments at adequate or luxury P supply (S3 Fig).

**Fig 3:**
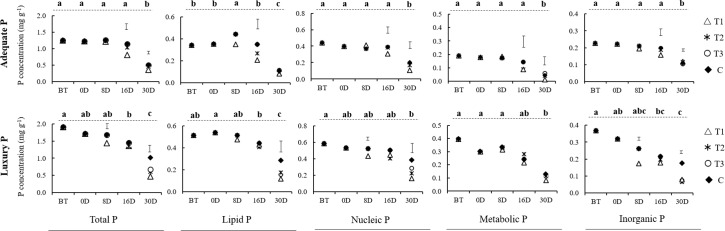
Effect of phosphorus removal treatments (T1 to T3) on total P concentration and concentration of lipids-P, nucleic-P, metabolic-P and inorganic P under adequate (top) or luxury (bottom) P supply. X-axis legend indicates the growth stage at which P concentrations were measured: booting (BT), anthesis (0D), 8DAA (8D), 16DAA (16D) and 30DAA (30D). Letters above the graph present statistical comparison of P concentrations between growth stages in the control plants (C, black diamonds). Means that do not share a common letter are significantly different at P ≤ 0.05 (n = 3). LSD bars are presented only when significant differences were observed between means of treatments T1, T2, T3 and the control C (P ≤ 0.05, n = 3).

At the booting stage (around 4 d before anthesis), total P concentration in the flag leaf was around 40% higher in the luxury P than adequate P treatment (around 1.8 vs 1.3 mg g^-1^; Fig 3). This difference was mainly reflected in the metabolic-P and Pi fractions, where P concentrations in the luxury P treatment were almost double those in the adequate P treatment. A similar pattern was also observed for the lipid-P and nucleic acid-P fractions that were the major P fractions under both adequate and luxury P supply.

Under luxury P supply a significant decline in total P, lipid-P and Pi was observed in control plants at 16 DAA. The withdrawal of P from the nutrient solution at anthesis caused a significant decline in total P concentration and the concentration of P in the nucleic acid-P and Pi fractions at 8 DAA, although there were no differences in P concentration among any P withdrawal treatments in any P fractions at 16 DAA. At maturity (30 DAA) the concentration of P in the lipid-P, nucleic acid-P and Pi fractions was significantly lower in treatment T1 than C. The concentration of P in the Pi fraction at 30 DAA was also significantly lower in treatment T2 than C (Fig 3).

Under adequate P supply, the concentration of leaf total P and P concentrations in all leaf P fractions in control plants only declined significantly at 30 DAA (Fig 3). The withdrawal of P from the nutrient solution at anthesis had no significant (P ≤ 0.05) impact on P concentrations in any leaf P fractions at 8 DAA, although lipid-P showed a substantial reduction of 20.5% (P = 0.07). However, significant reductions in P concentration of 40.7% (lipid-P), 24.9% (nucleic acid-P), 53.1% (metabolic-P) and 18.9% (Pi) were observed by 16 DAA (Fig 3). A significant 49.8% reduction in metabolic-P was also observed at 16 DAA when P was withdrawn from the nutrient solution at 8 DAA. Concentrations of P in all but the lipid-P and Pi factions were still significantly lower at maturity (30 DAA) when P was withdrawn from the nutrient solution at anthesis compared with that in control plants (Fig 3).

#### Effect on flag leaf photosynthesis

**Fig 4:**
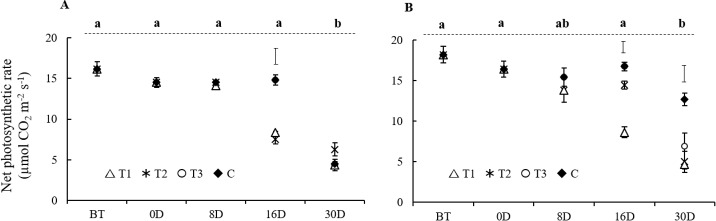
**Effect of P removal treatment (T1 to T3) on flag leaf photosynthetic rate under adequate P supply (A) and luxury P supply (B).** X-axis legend indicates the growth stage at which photosynthetic rate was measured: BT; booting, 0D; Anthesis, 8D; 8DAA, 16D; 16DAA and 30D; 30DAA. Letters above the graph are for statistical comparison of P concentrations in the control (C) treatment over time: C means (black diamonds) that do not share a common letter are significantly different of at P ≤ 0.05 (n = 3). Where a significant (P ≤ 0.05) difference between treatments T1-T3 and control (C) occurred at a given time point, LSD bars (P ≤ 5%) are presented to allow comparison between means. The absence of an LSD bar indicates that means were not significantly different at P ≤ 0.05.

At the booting stage (around 4 d before anthesis), photosynthetic rates were 16.17 ± 0.89 μmol CO_2_ m^-2^ s^-1^ under adequate P supply and 18.22 ± 1.02 μmol CO_2_ m^-2^ s^-1^ under luxury P supply, and there was no significant decline in these rates in control plants until beyond 16 DAA (Fig 4A and 4B). At maturity (30 DAA), however, photosynthetic rates in control plants had declined to 4.49 ± 0.29 μmol CO_2_ m^-2^ s^-1^ and 12.37± 0.77 μmol CO_2_ m^-2^ s^-1^ in plants under adequate and luxury P supply, respectively.

Under adequate P supply, the withdrawal of P from the nutrient solution at either anthesis or 8 DAA caused a significant reduction in photosynthesis (around 50%) at 16 DAA compared with that of control plants, while no differences were observed at maturity. In contrast, under luxury P supply the photosynthetic rate at 16 DAA had only declined significantly when P was withdrawn from the nutrient solution at anthesis (T1), and at maturity the photosynthetic rate in the control plants was significantly higher than the photosynthetic rates of plants in all other treatments (Fig 4B).

## Discussion

During senescence in grain crops, nutrients including P are remobilised from vegetative tissues with high efficiency to meet the demands of developing grains [8]. We hypothesised that the P demand of developing grains (P sink) would compete with the concurrent demands of photosynthetic tissues, and that competition for P during grain filling may be detrimental for photosynthesis with consequences for grain yields. We further hypothesised that to maintain photosynthesis, plants would remobilise P sequentially from the least to most essential P pools.

### Impact of restricted post-anthesis P supply on photosynthesis in flag leaves

Our hypothesis that restricting post-anthesis P uptake would lead to reductions in photosynthesis was supported by the data reported here. Indeed, while photosynthesis in the control treatment (C—P supplied in nutrient solution until maturity) declined significantly between 16 DAA and maturity at both adequate and luxury P supply, photosynthetic rates declined prematurely (at 16 DAA) when P was withdrawn from the nutrient solution at anthesis (T1) at luxury P supply, or at either anthesis (T1) or 8 DAA (T2) at adequate P supply (Fig 4). The reduction in photosynthetic rates between 16 DAA and maturity (30 DAA) in control plants is consistent with earlier reports [27] and coincides with the significant remobilisation of both total P (Fig 3) and total N (S4 Fig) from flag leaves between 16 DAA and 30 DAA. However, the decline in photosynthetic rate already observed at 16 DAA with the treatment T1 or T2 could not be attributed to remobilisation of N from flag leaves because N concentrations did not yet differ between treatments at 16 DAA at either adequate or luxury P supply (S4 Fig). Given that root uptake of P during grain filling contributes up to 70% of final rice (cv. IR64) grain P content [14], our data suggest that when exogenous P supply during grain filling is limited, the P demands of the developing seeds necessitate premature remobilisation of P from vegetative tissues, with consequent reductions in photosynthesis.

### Consequences of reduced photosynthesis on plant biomass and grain yields

Contrary to our hypothesis that any reductions in photosynthesis as a result of restricted post-anthesis P uptake would lead to yield reductions, competition for P between seeds and vegetative tissues under treatments T1 and T2 did not lead to a definitive reduction in biomass or grain yield despite the reductions in flag leaf photosynthetic capacity. In fact, grain yield was significantly higher under luxury P supply when P was withdrawn from the nutrient solution at anthesis (T1) than in all other treatment combinations (Table 1). We attribute this phenomenon to a lower number of late tillers in T1 (Table 1), when in other treatments a large portion of P was partitioned to these late tillers (Fig 2F). This reduction in late tiller number may also be an indication that P withdrawal at anthesis (after prior luxury P supply) induced plants to ‘focus’ on reproductive development. When P was withdrawn from the growing medium of *Brassica napus* during seed filling, the plant response was highly co-ordinated: when P was withdrawn at early flowering, plants aborted siliques, while when P was withdrawn during mid pod filling plant retained siliques but aborted seeds within siliques [13]. Flowering and seed set are tightly regulated, requiring the co-ordinated expression of many genes and physiological processes [28]. The concerted activity of several day length sensitive gene products inhibit flowering in rice [29] and when inhibition of stage in rice reproduction is released, a cascade of irreversible events and physiological processes are initiated which result in flowering and ultimately grain filling. The cell structures required for grain filling are achieved through a cell differentiation that is completed six days after flowering and followed by physiological maturity at 20 days after flowering [30, 31]. As a result of the tight regulation of the flowering and seed set processes, when P was withdrawn from the nutrient solution at anthesis or 8 DAA in the present work, plants did not invest energy or P in late tillers which enabled them to sustain or increase grain yield. Similarly, Mondal and Choudhuri [17] found that rice plants only allocated P to late tillers if panicles of the main tillers were excised, while P was preferentially allocated to grains of the main tillers if panicles were retained. Preferential allocation of P and carbon to grains at the expense of late tillers did not occur in the same treatment under adequate P supply because fewer late tillers per plant formed in any treatment owing to the lower P levels in plants. It therefore appears that grains are the main sink for P during senescence in rice, and investment in later forming tillers only occurs when excess P beyond the demands of the developing grains is available.

### Mobilisation of P fractions in flag leaves under restricted post-anthesis P supply

Total P concentrations and concentrations of P in flag leaf P fractions were always lower in the adequate P treatment compared with those in the luxury P treatment. One can therefore assume changes in specific P fractions have greater consequences in the adequate treatment, and our discussion of such changes will therefore focus predominantly on adequate treatment. The accumulation of P in developing rice grains follows a sigmoidal pattern, with a low rate of accumulation in the first week followed by a period of rapid accumulation between 7–16 DAA before tapering off beyond 16 DAA [15, 32]. Due to the lower P demands of the grain during the first week after anthesis, it was not surprising that neither concentration of total P nor P concentrations in any P fraction declined by 8 DAA when P supply was withheld at anthesis (Fig 3), and this was mirrored by unchanged net photosynthetic rates (Fig 4).

By 16 DAA concentrations of total P and concentrations in all P fractions had declined significantly in the adequate P treatment with a concomitant reduction in photosynthesis when P was withdrawn at anthesis. Lipid-P was the fraction with the most pronounced decline (-40%) and in fact this decline was already noticeable at 8 DAA (~20%; P = 0.07). Gene expression profiling during grain filling suggested the possible replacement of phospholipids with other lipids that do not contain P (e.g. sulfolipids) [16], and that the ~ 20% reduction in lipid-P concentrations by 8 DAA was not associated with concomitant reductions in photosynthesis also supports this notion. Similar changes have been observed in Proteaceae and this strategy is considered to be crucial sustaining photosynthesis under P-deficient conditions [33].

While early changes in lipid-P concentrations provide some support for our hypothesis that the least essential P pools would be mobilised earlier than the more critical P pools, the rather small and late changes in the inorganic P (Pi) pool seem contradictory since vacuolar-Pi is expected to be the most dispensable P pool. Here, a comparison with the luxury P treatment can help resolve this issue: the concentration of Pi was much greater in the luxury treatment compared with the adequate treatment, presumably indicating that luxury influx of P was stored as Pi in the vacuole. Upon withholding P supply, P concentrations in this fraction rapidly declined from 0.37 mg g^-1^ to below 0.2 mg g^-1^ and this reduction was the biggest change between T1 and other treatments observed across all P fractions at 8 DAA (Fig 3). It is entirely possible that the 0.2 mg g^-1^ P in the Pi fraction represents a minimum continuous Pi supply needed within the cell to reach maximum biomass and grain yields. Given that this concentration is at the lower limit of the 0.1–0.8 mg g^-1^ P range of metabolically active (cytosolic) Pi values reported in the literature [22], we hypothesise that this 0.2 mg g^-1^ P may represent metabolically active Pi in the cytosol rather than Pi stored in the vacuole. Ultimately, further specific studies of vacuolar Pi would be needed to confirm this hypothesis. The absence of a reduction in Pi fractions in the adequate P treatment may thus be a consequence of the lack of Pi stored in the vacuole together with the P supplied by the degradation of membrane phospholipids. The decline in flag leaf Pi concentrations in the luxury treatment lends further support to the hypothesis that remobilisation of P from senescing leaves during grain filling is co-ordinated to preserve leaf function for as long as possible.

When P was supplied until maturity (C) under luxury P supply, flag leaves were still green at maturity (S5 Fig) with lipid-P, nucleic-P, metabolic-P and Pi concentrations of around 0.3, 0.4, 0.13 and 0.2 mg g^-1^ P, respectively, and photosynthetic activity was still almost 15 μmol CO_2_ m^-2^ s^-1^. These concentrations are remarkably similar to the P concentrations observed in the respective P fractions in the control P treatment under adequate P supply from booting until 16 DAA (Fig 3), which suggests that such concentrations may be the minimum required for normal leaf function in rice flag leaves.

Our study supports the notion that competition for P between vegetative tissues and developing rice grains can impair photosynthesis when P supply during grain filling is limited, and that plants minimise photosynthetic reductions by remobilising less essential (e.g. vacuolar Pi) or replaceable (e.g. phospholipid-P) P pools prior to remobilisation of P in pools critical to leaf function such as nucleic acid-P and cytosolic Pi. In light of the competition between vegetative P requirements and the strong P sink strength of developing grains, any reduction in the P sink strength of grains by genetic manipulation may enable leaves to sustain high rates of photosynthesis until the later stages of grain filling.

## Supporting information

S1 Fig Schematic diagram of sequential phosphorus fractionation assay. * Indicates the tube needs to be dried under N air at the end of step. # indicates that the tube needs to be dried in rotational vacuum concentrator.
(PDF)

S2 Fig **Comparison of total P recovered by acid digestion and sum of all P fractions from sequential P fractionation in flag leaves of control treatments under A) adequate P supply; and B) luxury P supply.** Growth stages on x-axis defined as: BT; booting, 0D; Anthesis, 8D; 8DAA, 16D; 16DAA and 30D; 30DAA. Bars represent SEM (n = 3).
(PDF)

S3 Fig Proportion of P in each fraction in rice flag leaves under varying P supply and P withdrawal treatments. NP (nucleic P), LP (Lipid P), MP (Metabolic P), Pi (inorganic P) and RP (residual P).
(PDF)

S4 Fig **The impact of phosphorus withdrawal from the nutrient solution on flag leaf nitrogen concentrations under A) adequate P supply; and B) luxury P supply.** X-axis legend indicates the growth stage at which nitrogen concentrations were measured: BT; booting, 0D; Anthesis, 8D; 8DAA, 16D; 16DAA and 30D; 30DAA. Bars represent SEM (n = 3).
(PDF)

S5 Fig Photograph different treatment pots and individual panicles with flag leaves at harvest (30DAA). (PDF)
