# Supplementary figures and images for: Remobilisation of phosphorus fractions in rice flag leaves during grain filling: Implications for photosynthesis and grain yields
Source: PLoS One. 2017 Nov 2;12(11):e0187521. doi: 10.1371/journal.pone.0187521 (PMC5667883; doi:10.1371/journal.pone.0187521)

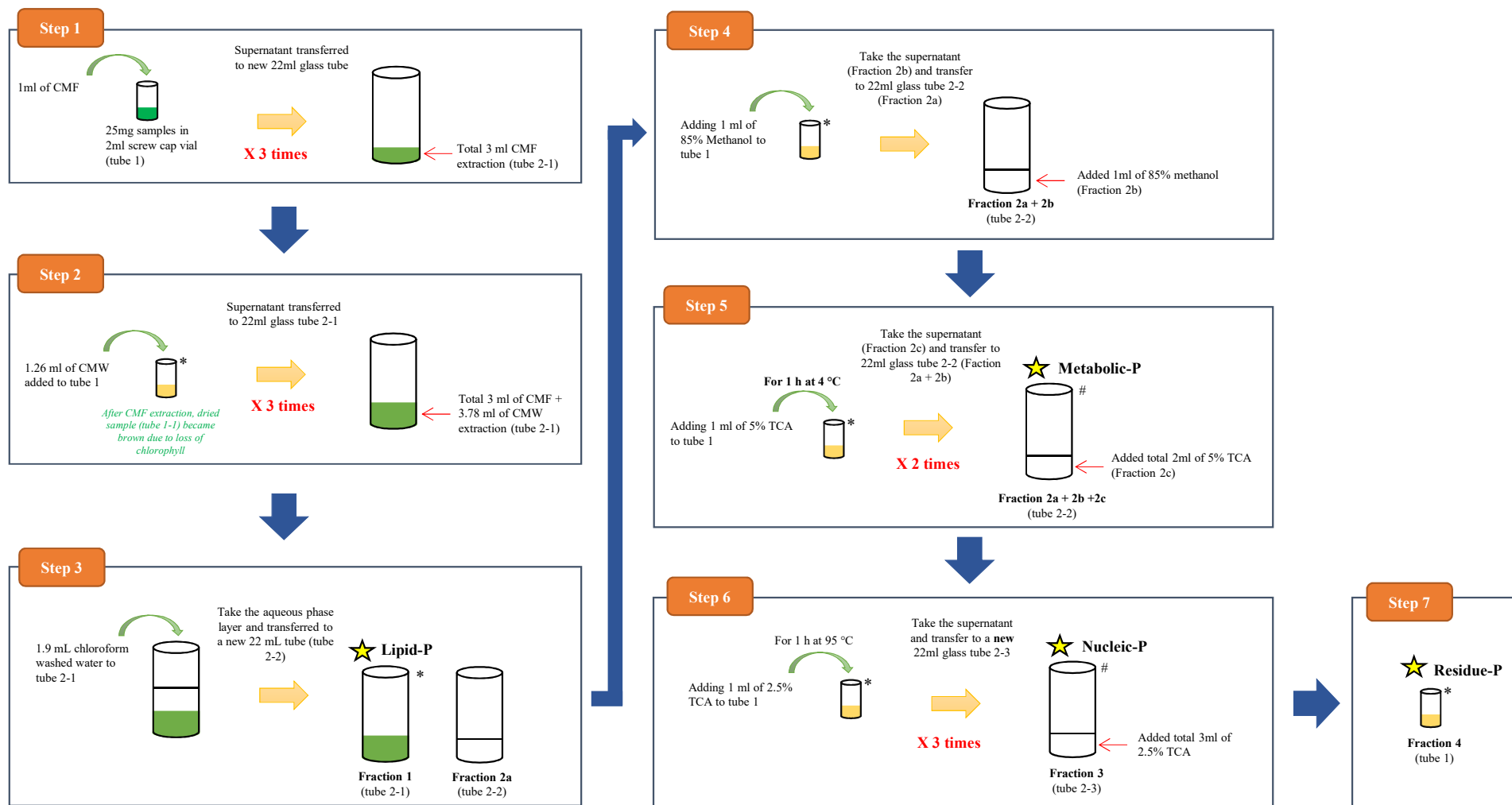

S1 Fig.

Supplement: S1 Fig — * Indicates the tube needs to be dried under N air at the end of step. # indicates that the tube needs to be dried in rotational vacuum concentrator. (PDF) [file pone.0187521.s001.pdf]

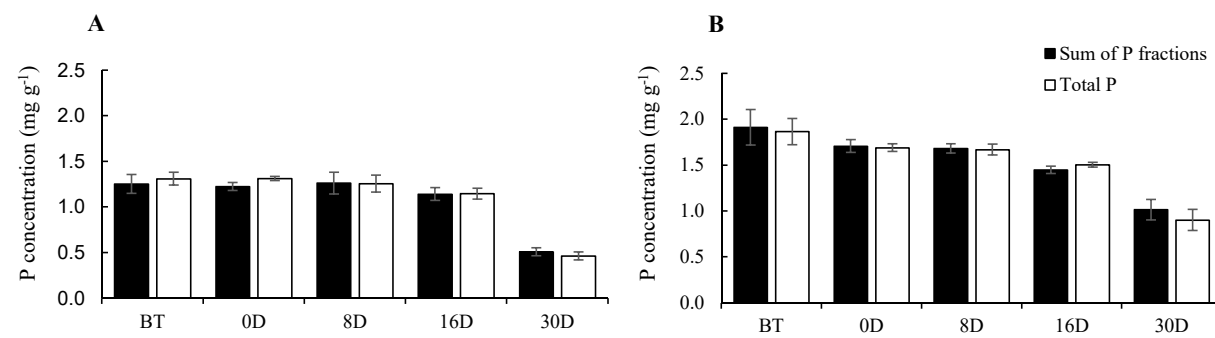

**S2 Fig.**

Supplement: S2 Fig — Comparison of total P recovered by acid digestion and sum of all P fractions from sequential P fractionation in flag leaves of control treatments under A) adequate P supply; and B) luxury P supply. Growth stages on x-axis defined as: BT; booting, 0D; Anthesis, 8D; 8DAA, 16D; 16DAA and 30D; 30DAA. Bars represent SEM (n = 3). (PDF) [file pone.0187521.s002.pdf]

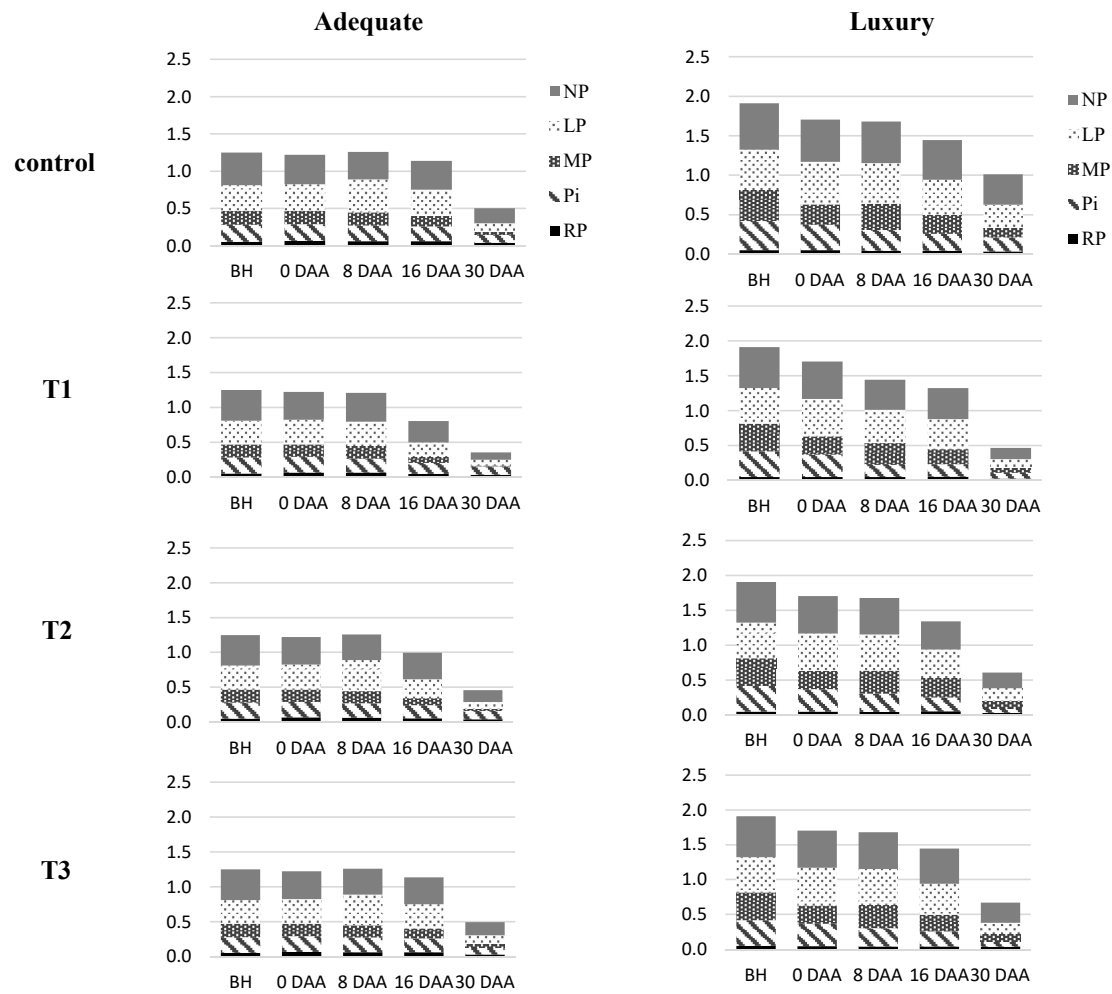

S3 Fig.

Supplement: S3 Fig — NP (nucleic P), LP (Lipid P), MP (Metabolic P), Pi (inorganic P) and RP (residual P). (PDF) [file pone.0187521.s003.pdf]

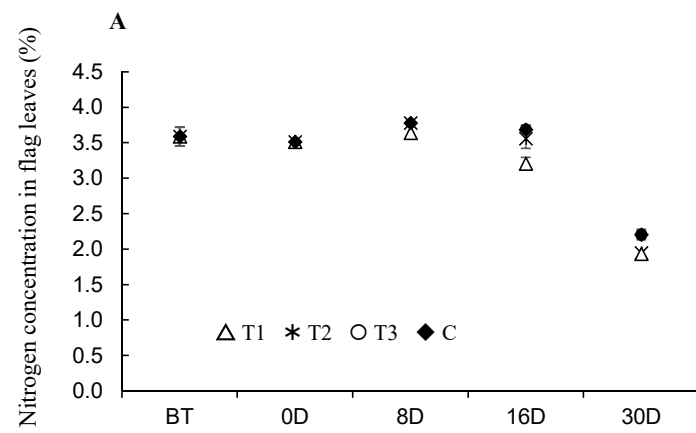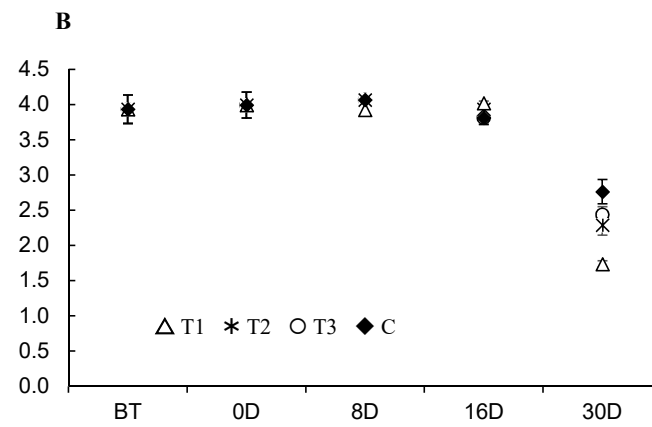

**S4 Fig.**

Supplement: S4 Fig — The impact of phosphorus withdrawal from the nutrient solution on flag leaf nitrogen concentrations under A) adequate P supply; and B) luxury P supply. X-axis legend indicates the growth stage at which nitrogen concentrations were measured: BT; booting, 0D; Anthesis, 8D; 8DAA, 16D; 16DAA and 30D; 30DAA. Bars represent SEM (n = 3). (PDF) [file pone.0187521.s004.pdf]

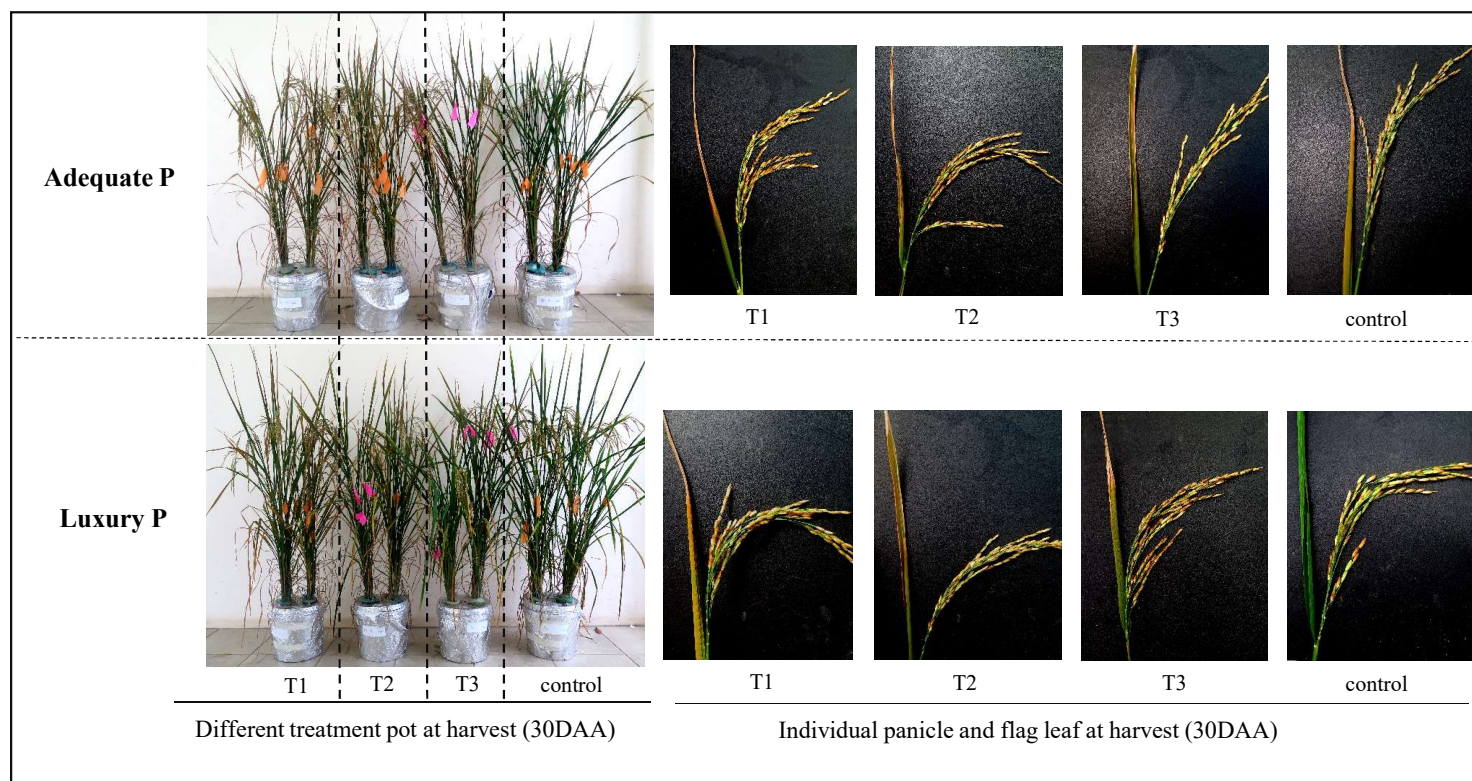

**S5 Fig.**

Supplement: S5 Fig — (PDF) [file pone.0187521.s005.pdf]
